# Safety, immunogenicity, and long COVID outcomes following inactivated COVID-19 vaccine boosters in elderly Chinese: a prospective cohort study

**DOI:** 10.3389/fimmu.2026.1813716

**Published:** 2026-04-30

**Authors:** Rongrong Dai, Qianhui Hua, Xinyu Liu, Lanxin Ma, Ruijing Bao, Huijun Lei, Pengfei Yu, Yuting Liao, Juan Yang, Su Han, Jianmin Jiang, Hangjie Zhang

**Affiliations:** 1 Department of Public Health and Preventive Medicine, Wuxi School of Medicine, Jiangnan University, Wuxi, China; 2 School of Public Health, Hangzhou Medical College, Hangzhou, China; 3 Department of Prevention and Control of Infectious Disease, Zhejiang Provincial Center for Disease Control and Prevention, Hangzhou, China; 4 School of Life Sci & Agr Forestry, Qiqihar University, Qiqihar, China; 5 Longyou District Center for Disease Control and Prevention, Quzhou, China; 6 Department of Immunization Program, Jiaxing City Center for Disease Control and Prevention, Jiaxing, China; 7 Zhejiang Key Laboratory of Vaccine, Infectious Disease Prevention and Control, Zhejiang Provincial Center for Disease Control and Prevention, Hangzhou, China

**Keywords:** breakthrough infection, dynamic changes, humoral immune response, inactivated COVID-19 vaccine, SARS-CoV-2, vaccine

## Abstract

**Background:**

Adults aged ≥60 years face elevated risks of severe COVID-19 and long COVID. Although inactivated SARS-CoV-2 vaccines are safe and effective, critical gaps remain regarding optimal booster timing, durability of immune responses, and protection against emerging variants in this vulnerable population.

**Methods:**

In this prospective cohort study in Zhejiang Province, China, 450 adults aged ≥60 years were randomized to receive an inactivated SARS-Cov-2 vaccine (CoronaVac or Covilo) under three dosing schedules. Neutralizing antibody titers, SARS-CoV-2-specific IgG, and variant-specific neutralization were evaluated using serum samples collected at multiple timepoints. Safety outcomes included local and systemic adverse reactions and long COVID symptoms.

**Results:**

Participants (age: 60–80 years; 50.9% male) with balanced baseline demographics were stratified into six subgroups by vaccine type and schedule (S1/S2/S3; n=75 each; all P>0.05). Adverse reaction incidence was low (0.0%–8.0%), with CoronaVac-S3 recipients experiencing significantly more local reactions than Covilo-S3 recipients (8.0% vs. 0.0%; P = 0.0124). Immunogenicity varied markedly at 1 month post vaccination, with the highest geometric mean titers (GMTs) of neutralizing antibodies in both S3 groups (Covilo: 57.2; CoronaVac: 59.4; P<0.001 vs. respective S1/S2 groups), and CoronaVac-S1 inducing higher GMTs than Covilo-S1 (21.8 vs. 15.4; P = 0.009). At 12 months post vaccination, GMTs remained highest in Covilo-S3 (9.9), while CoronaVac-S2 exceeded Covilo-S2 (8.0 vs. 5.1; P = 0.021). Covilo-S2 and -S3 were superior to their CoronaVac counterparts in inhibition against the wild-type strain and Delta variant at 1 month post vaccination (all P<0.05). Multivariate analysis identified male sex as a protective factor against COVID-19 symptoms, long COVID, and fatigue (odds ratios <0.5 and P<0.05 for all).

**Conclusions:**

A third booster of inactivated SARS-CoV-2 vaccine administered within 2–6 months of the second dose is safe and significantly boosts humoral immunity in adults ≥60 years. The three-dose Covilo regimen and a 6-month dosing interval optimized immunogenicity and were associated with the lowest risks of COVID-19 symptoms and long COVID, highlighting the importance of vaccine-specific and interval-adjusted booster strategies for older populations.

## Introduction

The emergence and global spread of coronavirus disease 2019 (COVID-19), caused by severe acute respiratory syndrome coronavirus 2 (SARS-CoV-2), remains a major public health challenge (1, 2). Adults aged ≥60 years face heightened SARS-CoV-2 infection risks related to physiological decline, chronic comorbidities, and compromised immune function, which collectively contribute to increased disease severity, complications, and mortality (3, 4).

There is accumulating evidence that SARS-CoV-2 vaccination confers protective effects in this population (5, 6). Inactivated SARS-CoV-2 vaccines such as CoronaVac and Covilo are widely used domestically and internationally, with several clinical trials demonstrating their general safety and efficacy in older populations (7). Specifically, in adults aged ≥60 years, CoronaVac and Covilo have achieved seroconversion rates of 98% and 100%, respectively, with adverse reactions predominantly ranging from mild to moderate in severity (8, 9). However, in observational studies from Malaysia and China, vaccine effectiveness against infection declined significantly within 3–6 months after primary immunization. This trend is consistent across both mRNA (e.g., BNT162b2) and inactivated vaccines. CoronaVac has also shown reduced protection against severe outcomes, such as intensive care unit admission (10, 11). Multiple diverse studies reported waning immunity within 2–6 months post primary immunization (12–14), particularly in older adults, who may face nearly twice the risk of severe COVID-19 after this decline (15–17). Current evidence suggests that vaccination intervals modulate the magnitude and persistence of vaccine-induced immunity. Administering booster doses within this 2- to 6-month window helps sustain adequate immune protection, which is of paramount importance for elderly and high-risk populations (18).

This challenge is further compounded by SARS-CoV-2 genetic diversity, with emerging variants exhibiting reduced susceptibility to neutralizing antibodies elicited by primary vaccination (19, 20). Despite widespread vaccination, continuous viral evolution, exemplified by the Omicron variant, has enhanced the ability of the virus to evade neutralizing antibodies, resulting in breakthrough infections (17, 21).

Post-acute sequelae of COVID-19 or long COVID has emerged as a persistent global public health challenge, affecting millions worldwide (22). As defined by the World Health Organization, long COVID refers to new, recurring, or persistent symptoms that develop within 3 months of an initial, acute SARS-CoV-2 infection and last for at least 2 months, encompassing over 200 manifestations (e.g., fatigue, dyspnea, cognitive impairment, musculoskeletal pain) that impair daily functioning and quality of life (22). Accumulating evidence indicates that long COVID can occur regardless of acute disease severity, but older and immunocompromised individuals face heightened risk attributable to age-related immunosenescence, inflammaging, and comorbidities (23).

Following the adjustment of China’s COVID-19 prevention and control policies in late 2022, the country experienced a large-scale wave of first infections. During that period, older and immunocompromised individuals were exposed not only to heightened risks of acute infection but also to a markedly increased likelihood of developing long COVID (24–26).

Here, we conducted a prospective cohort investigation of 450 adults aged ≥60 years, assigned to one of three schedules of an inactivated SARS-CoV-2 vaccine (Covilo or CoronaVac), with 12-month follow-up to evaluate vaccine immunogenicity, safety, and protective efficacy against acute infection and long COVID. The aim was to strengthen clinical evidence for inactivated vaccines in older populations and provide a scientific basis for refining targeted immunization strategies.

## Results

### Demographic characteristics

A total of 450 participants were randomized into three groups (S1–S3, n=150/group) based on vaccination schedule (S1, S2, S3) (**Table 1**). Each group received either the Covilo or CoronaVac vaccine, resulting in six subgroups with 75 participants each. Among all participants (age: 60–80 years), 229 (50.9%) were male and 221 (49.1%) were female, with no significant difference in sex (P = 0.943) or age (P = 0.201) distribution among the subgroups. Body mass index (BMI) was <24.0 in 303 participants (67.3%) and ≥24.0 in 147 (32.7%). Chi-square tests and one-way analysis of variance (ANOVA) confirmed that there were no significant differences in BMI (P = 0.084) among the subgroups.

**Table 1:** Demographic characteristics of the study participants.

Variable	Covilo			CoronaVac			
	S1(N = 75)	S2(N = 75)	S3(N = 75)	S1(N = 75)	S2(N = 75)	S3(N = 75)	p
Gender							0.943
Female	34(45.3)	35(46.7)	37(49.3)	38(50.7)	37(49.3)	40(53.3)	
Male	41(54.7)	40(53.3)	38(50.7)	37(49.3)	38(50.7)	35(46.7)	
Age, years							0.201
60-69	45(60.0)	46(61.3)	52(70.3)	44(58.7)	44(58.7)	55(73.3)	
70-80	30(40.0)	29(38.7)	22(29.7)	31(41.3)	31(41.3)	19(25.3)	
IQR	68(64, 71)	67(64, 72)	68(65, 71)	68(65, 73)	69(64, 71)	65(63, 70)	
BMI							0.084
<24.0	47(62.7)	52(69.3)	44(58.7)	53(70.7)	48(64.0)	60(80.0)	
≥24.0	28(37.3)	23(30.7)	31(41.3)	22(29.3)	27(36.0)	15(20.0)	

Data expressed as the number of participants (% or median [P_25_, P_75_]). S1, S2, and S3 represent three different vaccination schedules: S1: two doses administered one month apart; S2: three doses each administered one month apart; S3: three doses with the first two doses given one month apart and an interval of 4–6 months between the second and third doses. Age data were missing for one participant in S1.

### Safety analysis

The overall incidence of vaccine-related adverse reactions was low across all subgroups (0.0%–8.0%; **Table 2**). Adverse reactions were reported in 0.0% of Covilo-S1 and -S3, 5.3% of Covilo-S2, 1.3% in CoronaVac-S1 and -S2, and 8.0% of CoronaVac-S3. Notably, within the S3 group, CoronaVac recipients had a significantly higher incidence than Covilo recipients (8.0% vs. 0.0%; P = 0.0124).

**Table 2:** Adverse reactions occurring within 7 days after each vaccine dose.

Variable	Covilo				CoronaVac			
	S1(N = 75)	S2(N = 75)		S3(N = 75)	S1(N = 75)	S2(N = 75)	S3(N = 75)	p
Total adverse reactions								0.008
No	75(100.0)		71(94.7)	75(100.0)	74(98.7)	74(98.7)	69(92.0)	
Yes	0(0.0)		4(5.3)	0(0.0)	1(1.3)	1(1.3)	6(8.0)	
Systemic adverse reactions								0.999
No	75(100.0)		74(98.7)	75(100.0)	75(100.0)	75(100.0)	74(98.7)	
Yes	0(0.0)		1(1.3)	0(0.0)	0(0.0)	0(0.0)	1(1.3)	
Allergy	0(0.0)		1(1.3)	0(0.0)	0(0.0)	0(0.0)	0(0.0)	0.999
Fever	0(0.0)		0(0.0)	0(0.0)	0(0.0)	0(0.0)	1(1.3)	0.999
Local adverse events								0.003
No	75(100.0)		72(96.0)	75(100.0)	75(100.0)	74(98.7)	69(92.0)	
Yes	0(0.0)		3(4.0)	0(0.0)	0(0.0)	1(1.3)	6(8.0)	
Pain	0(0.0)		3(4.0)	0(0.0)	0(0.0)	1(1.3)	6(8.0)	0.003
Flush	0(0.0)		0(0.0)	0(0.0)	0(0.0)	0(0.0)	3(4.0)	0.027

Data expressed as the number of participants experiencing the event (%). Each participant was counted only once within each specific reaction category, even if they experienced multiple adverse reactions. Adverse reactions and events were graded using a scale issued by the China State Food and Drug Administration.

Systemic adverse reactions were limited to the Covilo-S2 (allergy, 1.3%) and CoronaVac-S3 (fever, 1.3%) subgroups. Local adverse reactions were also infrequent (0.0%–8.0%), occurring in 4.0% of Covilo-S2, 1.3% of CoronaVac-S2, and 8.0% of CoronaVac-S3 recipients. Within the S3 group, local reactions were significantly more common with CoronaVac (8.0% vs. 0.0%; P = 0.0124). Pain was reported in Covilo-S2 (4.0%), CoronaVac-S2 (1.3%), and CoronaVac-S3 (8.0%), while flush was only reported in CoronaVac-S3 (4.0%). Overall, the safety profiles of the two vaccines were generally comparable across the different schedules, with adverse reactions being uncommon.

### Analysis of immunogenicity and associated factors

#### SARS-CoV-2 neutralizing antibodies

**Figure 1:**
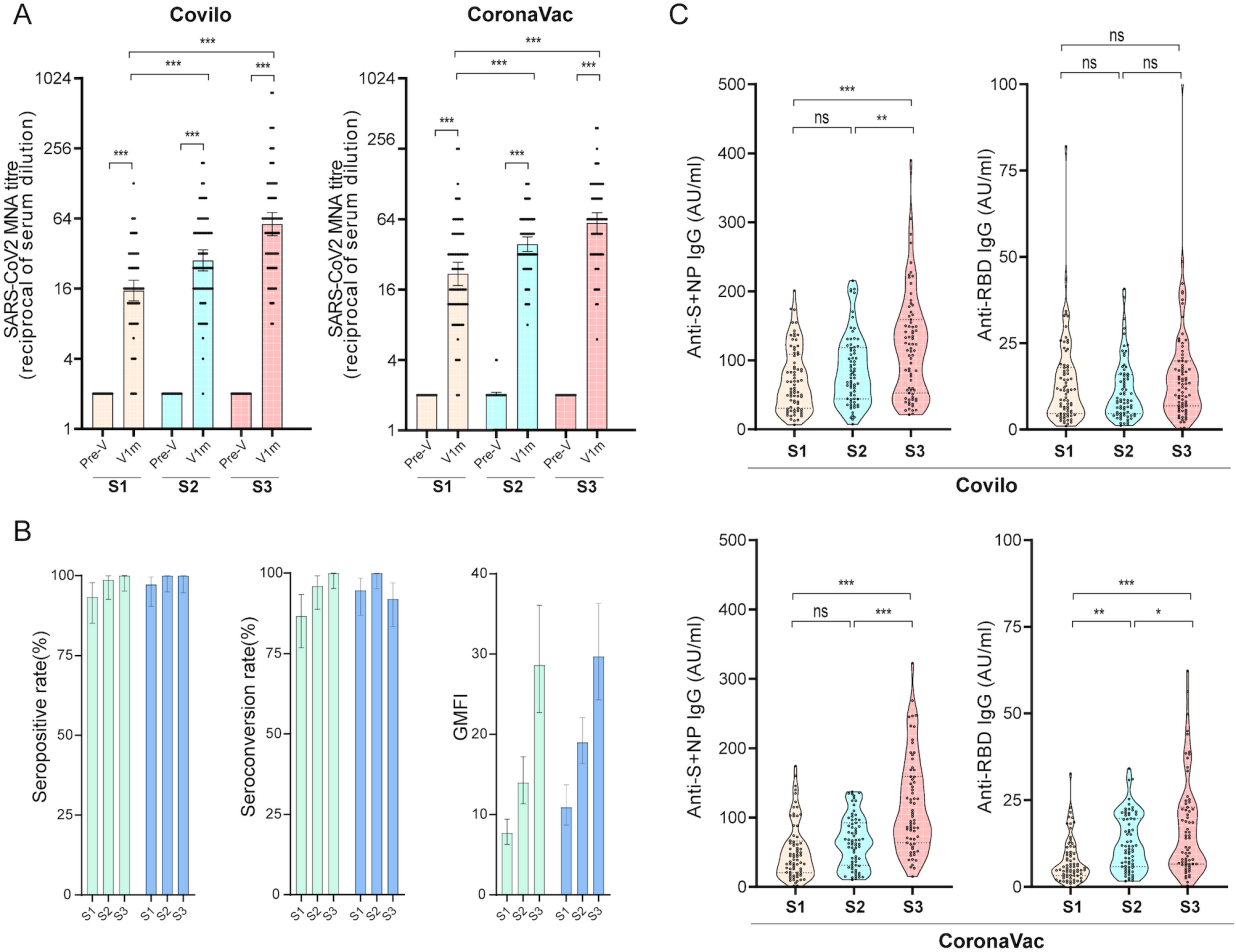
Antibody responses at baseline and 1 month after the second or third vaccine dose. Neutralizing antibody titers **(A)**, seropositivity and seroconversion rates (%), and geometric mean fold increases (GMFI) of neutralizing antibodies (NAbs) **(B)**, anti-S&N IgG, and anti-RBD IgG **(C)** against SARS-CoV-2 (Wuhan strain) were measured at baseline (T0) and 28 days (T1) after the second or third dose of inactivated COVID-19 vaccines (CoronaVac or Covilo) under indistinct immunization schedules. Data are expressed as geometric means ± 95% confidence intervals (95% CI). Neutralizing antibody positivity was defined as a titer ≥ 1:8. Seroconversion was defined as a fourfold increase in antibody concentration post-vaccination compared with pre-vaccination levels. Statistical significance was assessed using two-tailed Student’s t-test and one-way analysis of variance (ANOVA) followed by Tukey’s multiple comparisons test. P-values < 0.05 were considered statistically significant. 95% CI, 95% confidence interval; GMT, geometric mean titer; GMC, geometric mean concentration; GMFI, geometric mean fold increase; MNA, microneutralization assay. ns, not significant; *P<0.05, **P<0.01, ***P<0.001.

At baseline (T0), the geometric mean titer (GMT) of SARS-CoV-2 neutralizing antibodies was 2.0 (95% confidence interval [CI]: 2.0, 2.0) across all groups, with no significant between-group differences (**Figure 1A**, **Table 3**). At 1 month post vaccination (T1), GMTs were 15.4 (12.6, 18.9) in Covilo-S1, 27.9 (22.7, 34.4) in Covilo-S2, and 57.2 (45.4, 72.2) in Covilo-S3, with pairwise comparisons revealing significant between-group differences (P<0.001). The CoronaVac subgroups also showed significant between-group differences (P<0.001), with GMTs of 21.8 (17.3, 27.4) in CoronaVac-S1, 39.2 (33.9, 45.4) in CoronaVac-S2, and 59.4 (48.6, 72.7) in CoronaVac-S3. Within the S1 group, these values were significantly higher in CoronaVac recipients than in Covilo recipients (P = 0.009). Seropositivity at this timepoint ranged from 93.3% to 100.0% across all groups. At T1, the geometric mean fold increase (GMFI) in SARS-CoV-2 neutralizing antibodies was 7.7 (95% CI: 6.3, 9.4), 14.0 (11.3, 17.2), and 28.6 (22.7, 36.1) in Covilo-S1, -S2, and -S3, respectively, and 10.9 (8.7, 13.7), 19.0 (16.3, 22.1), and 29.7 (24.3, 36.3) in the corresponding CoronaVac subgroups, with pairwise comparisons revealing significant between-group differences (P<0.001). Within the S2 group, the GMFI values were significantly higher in CoronaVac recipients than in Covilo recipients (P = 0.018).

**Table 3:** Neutralizing antibodies against SARS-CoV-2 in the vaccine subgroups.

Time point		Covilo			CoronaVac			p
T0	Groups	**S1(N = 75)**	**S2(N = 75)**	**S3(N = 75)**	**S1(N = 75)**	**S2(N = 75)**	**S3(N = 75)**	
	GMT	2.0(2.0,2.0)	2.0(2.0,2.0)	2.0(2.0,2.0)	2.0(2.0,2.0)	2.1(2.0,2.1)	2.0(2.0,2.0)	1.000
	Seropositive rate (%)	0.0(0.0,4.8)	0.0(0.0,4.8)	0.0(0.0,4.8)	0.0(0.0,4.8)	4.0(0.9,11.5)	0.0(0.0,4.8)	0.004
T1	Groups	**S1(N = 75)**	**S2(N = 73)**	**S3(N = 75)**	**S1(N = 73)**	**S2(N = 71)**	**S3(N = 67)**	
	GMT	15.4(12.6,18.9)	27.9(22.7,34.4)	57.2(45.4,72.2)	21.8(17.3,27.4)	39.2(33.9,45.4)	59.4(48.6,72.7)	<0.001
	Seropositive rate (%)	93.3(85.1,97.8)	98.6(92.6,100.0)	100.0(95.2,100.0)	97.3(90.5,99.7)	100.0(94.9,100.0)	100.0(94.6,100.0)	0.014
	Seroconversion rate(%) e (%)	86.7(76.8,93.4)	96.0(88.8,99.2)	100.0(95.2,100.0)	94.7(86.9,98.5)	100.0(95.2,100.0)	92.0(83.4,97.0)	<0.001
	GMFI	7.7(6.3,9.4)	14.0(11.3,17.2)	28.6(22.7,36.1)	10.9(8.7,13.7)	19.0(16.3,22.1)	29.7(24.3,36.3)	<0.001
T2	Groups	**S1(N = 73)**	**S2(N = 73)**	**S3(N = 49)**	**S1(N = 63)**	**S2(N = 70)**	**S3(N = 59)**	
	GMT	7.5(6.0,9.4)	8.8(7.5,10.2)	13.1(10.3,16.7)	4.4(3.7,5.3)	10.4(8.9,12.2)	19.7(15.9,24.4)	<0.001
	Seropositive rate (%)	82.2(71.5,90.2)	93.2(84.7,97.7)	93.9(83.1,98.7)	66.7(53.7,78)	95.7(88,99.1)	98.3(90.9,100.0)	<0.001
T3	Groups	**S1(N = 34)^a^**	**S2(N = 71)**	**S3(N = 73)**	**S1(N = 0)^a^**	**S2(N = 69)**	**S3(N = 60)**	
	GMT	4.0(2.7,5.7)	5.7(4.7,6.9)	14.6(11.9,17.8)	–	5.9(4.9,7.1)	14.1(11.6,17.2)	<0.001
	Seropositive rate (%)	44.1(27.2,62.1)	74.6(62.9,84.2)	97.3(90.5,99.7)	–	78.3(66.7,87.3)	98.3(91.1,100.0)	<0.001
T4	Groups	**S1(N = 33)**	**S2(N = 70)**	**S3(N = 73)**	**S1(N = 0)**	**S2(N = 66)**	**S3(N = 53)**	
	GMT	3.7(2.8,4.9)	5.1(4.2,6.3)	9.9(7.8,12.6)	–	8.0(6.2,10.3)	10.0(7.7,13.0)	<0.001
	Seropositive rate (%)	48.5(30.8,66.5)	64.3(51.9,75.4)	83.6(73.0,91.2)	–	83.3(72.1,91.4)	88.7(77.0,95.7)	<0.001

Data presented as geometric mean titer (GMT) or geometric mean fold increase (GMFI) with the 95% confidence interval in parentheses. Positivity was defined as a neutralizing antibody value > 1:8. Seroconversion was defined as a fourfold increase in post-immunization concentration compared with the pre-immunization level. A “-” sign indicates mean loss to follow-up. ^a^Some or all participants were required to receive a third vaccine dose to remain in compliance with changing local COVID-19 vaccination policy. Bold values indicate the number of participants (N) in each subgroup.

**Figure 2:**
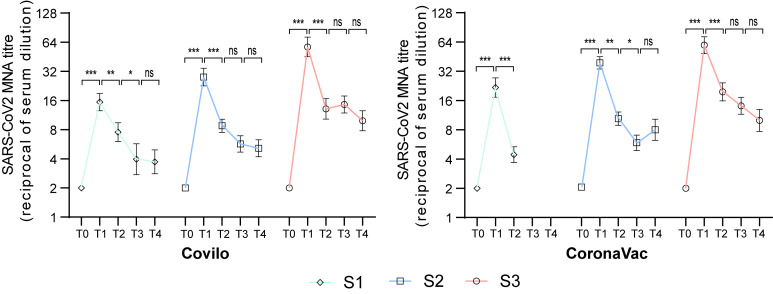
Longitudinal dynamics of neutralizing antibodies under distinct immunization schedules. Longitudinal dynamics of neutralizing antibodies against SARS-CoV-2 (Wuhan strain) in serum samples from participants aged ≥60 years vaccinated with CoronaVac or Covilo were analyzed at predefined time points: prior to the first dose (T0); and 1 (T1), 6 (T2), 9 (T3), and 12 (T4) months after completion of the assigned vaccination schedule. Data are expressed as geometric means ± 95% confidence intervals (95% CI). Neutralizing antibody positivity was defined as a titer ≥ 1:8. P values < 0.05 were considered statistically significant. 95% CI, 95% confidence interval; MNA, microneutralization assay. ns, not significant; *P<0.05, **P<0.01, ***P<0.001.

At 6 months post vaccination (T2), GMTs were 7.5 (6.0, 9.4), 8.8 (7.5, 10.2), and 13.1 (10.3, 16.7) in Covilo-S1, -S2, and -S3, respectively (P = 0.001), and were 4.4 (3.7, 5.3), 10.4 (8.9, 12.2), and 19.7 (15.9, 24.4) in CoronaVac-S1, -S2, and -S3, respectively (P<0.001). GMT values were significantly higher in Covilo-S1 recipients than in CoronaVac-S1 recipients (P = 0.001), and in CoronaVac-S3 recipients than in Covilo-S3 recipients (P = 0.017). Seropositivity at this timepoint ranged from 66.7% to 98.3% across all groups (**Figures 1A**, **2**, **Table 3**).

At 9 months post vaccination T3, GMTs were 4.0 (2.7, 5.7), 5.7 (4.7, 6.9), and 14.6 (11.9, 17.8) in Covilo-S1, -S2, and -S3 (P<0.001). For CoronaVac, GMTs were 10.4 (8.9, 12.2) in S2 and 19.7 (15.9, 24.4) in S3 (P<0.001). Seropositivity at this timepoint ranged from 44.1% to 98.3% across all groups (**Figures 1A**, **2**, **Table 3**).

At 12 months post vaccination (T4), GMTs were 3.7 (2.8,.9), 5.1 (4.2, 6.3), and 9.9 (7.8, 12.6) in Covilo-S1, -S2, and -S3, respectively, with pairwise comparisons revealing significant between-group differences (P<0.001). In contrast, there were no significant differences (P = 0.094) in GMTs between CoronaVac-S2 and CoronaVac-S3 at 8.0 (6.2, 10.3) and 10.0 (7.7, 13.0), respectively. Within the S2 group, GMT values were significantly higher in CoronaVac recipients than in Covilo recipients (P = 0.021). Seropositivity ranged from 48.5% to 88.7% across all groups at this timepoint (**Figures 1A**, **2**, **Table 3**).

#### SARS-CoV-2 anti-S1+NP IgG

Comprehensive analysis (**Figure 1C**) of IgG antibodies against the SARS-CoV-2 spike protein subunit S1 and nucleocapsid protein (anti-S1+NP) at T1 in 434 participants revealed seropositivity in 427 (98.4%). Specifically, 74/75 (98.7%) in Covilo-S1, 72/73 (98.6%) in Covilo-S2, and all 75 participants (100.0%) in Covilo-S3 tested positive, with no significant between-group differences (P = 0.773). Similarly, 68/73 (93.2%) in CoronaVac-S1, all 71 in CoronaVac-S2 (100.0%), and all 67 participants in the CoronaVac-S3 subgroup (100.0%) were seropositive (P = 0.011).

Notably, the geometric mean concentration (GMC) of anti-S1+NP antibodies at T1 was 57.0 AU/mL (95% CI: 48.1, 67.5) in Covilo-S1, 70.3 AU/mL (60.0, 82.6) in Covilo-S2, and 96.1 AU/mL (81.7, 113.0) in Covilo-S3. Compared with Covilo-S3, the Covilo-S1 and Covilo-S2 subgroups showed significant differences in GMC (P<0.001 and P = 0.025, respectively). For CoronaVac, GMCs were 36.4 AU/mL (29.1, 45.6) in CoronaVac-S1, 53.1 AU/mL (45.0, 62.8) in CoronaVac-S2, and 98.8 AU/mL (84.6, 115.5) in CoronaVac-S2, with S3 significantly higher than S1 and S2 (both P<0.001).

#### SARS-CoV-2 anti-RBD IgG

Testing for IgG antibodies against the receptor-binding domain (RBD) of SARS-Co-V-2 spike protein (anti-RBD IgG) was undertaken in 434 participants, with 428 (98.6%) testing positive (**Figure 1C**). Specifically, 74/75 (98.7%) participants in Covilo-S1, 73/73 (100%) in Covilo-S2, 73/75 (97.3%) in Covilo-S3 were positive, with no significant between-group differences (P = 0.775). For CoronaVac, 71/73 (97.3%) in CoronaVac-S1, 71/71 (100%) in CoronaVac-S2, and 67/68 (98.5%) participants in CoronaVac-S3 were positive, with no significant between-group differences (P = 0.540). GMCs were 9.6 AU/mL (95% CI: 7.8, 11.7), 8.5 AU/mL (7.1, 10.3), and 11.1 AU/mL (8.9, 13.8) in Covilo-S1, -S2, and -S3, respectively, with no significant between-group differences (P = 0.103). For CoronaVac, GMCs were 5.3 AU/mL (4.1, 6.6), 9.9 AU/mL (8.3, 11.9), and 11.9 AU/mL (9.4, 15.0) in S1, S2, and S3, with S2 and S3 significantly higher than S1 (both P<0.001).

### Infection inhibition percentage of SARS-CoV-2 WT, delta, and omicron

**Figure 3:**
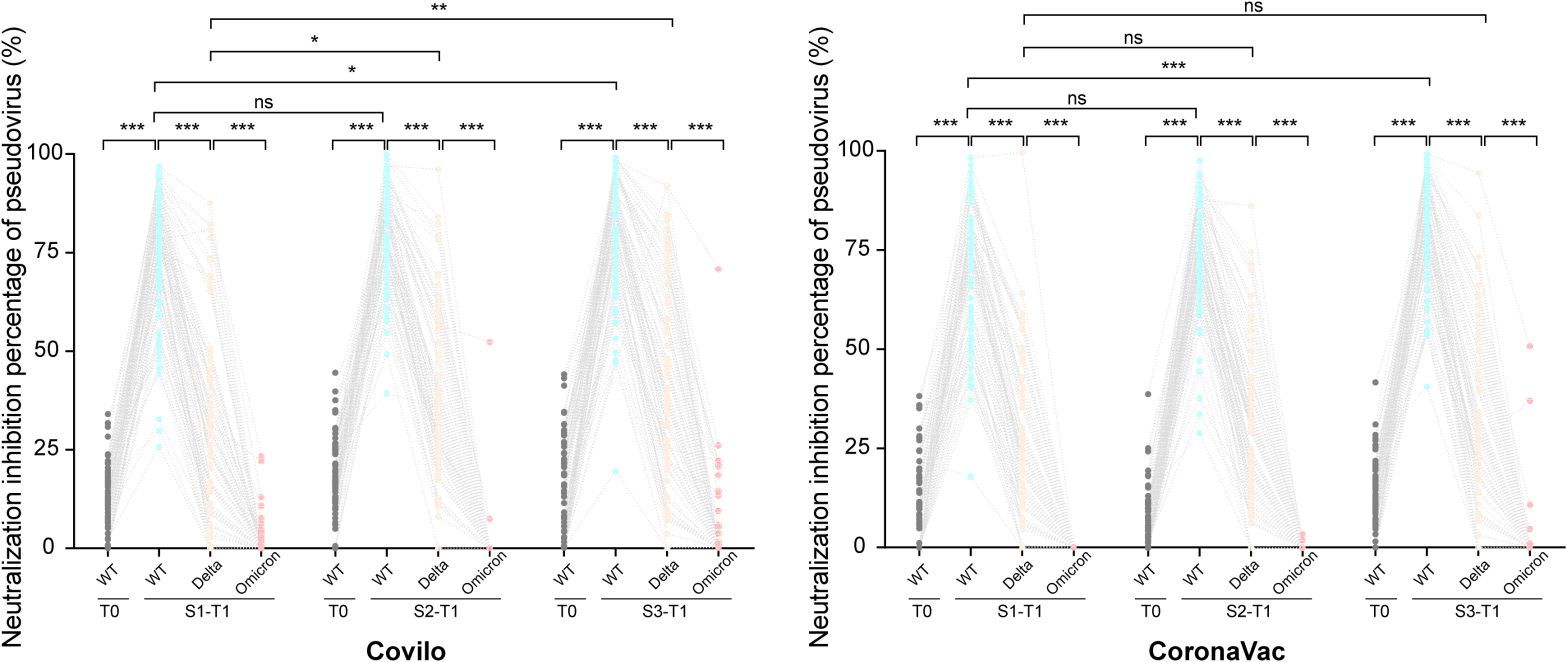
Antibody responses against SARS-CoV-2 delta and omicron variants. Neutralization activity against the SARS-CoV-2 Wuhan strain, Delta variant (B.1.617.2), and Omicron variant (B.1.1.529) was measured at baseline (T0) and 1 month (T1) following the second or third dose of inactivated COVID-19 vaccines (CoronaVac or Covilo) under indistinct immunization schedules, as assessed via pseudovirus-based neutralization assays. The paired Student’s t-test and one-way analysis of variance were used for comparison, with two-tailed P values computed for significance testing. *P < 0.05, **P < 0.01, ***P < 0.001.

**Figure 3** illustrates inhibition percentage against infection with WT, Delta, and Omicron variants across the vaccination groups. Before vaccination (T0), WT inhibition percentage were 13.0% (95% CI: 6.5, 19.0), 16.2% (12.8, 24.2), and 11.4% (0.5, 23.8) in Covilo-S1, -S2, and -S3, respectively, with significant differences between the S2 subgroup and S1 (P = 0.031) and S3 (P = 0.013). For CoronaVac, WT inhibition percentage were 9.8% (0.0, 18.2) in S1, 2.9% (0.0, 9.9) in S2, and 12.0% in S3 (5.4, 17.1), with significant differences between the S2 subgroup and S1 (P = 0.006) and S3 (P<0.001). Furthermore, infection inhibition percentage were significantly higher for the S2 recipients of Covilo than for CoronaVac (P<0.001).

At T1, WT inhibition percentage increased significantly in all groups compared with baseline, reaching 77.4% (66.7, 86.8), 81.5% (72.0, 88.3), and 83.8% (71.2, 92.8) in Covilo-S1, -S2, and -S3, respectively, with a significant difference between S1 and S3 (P = 0.033). For CoronaVac, rates were 73.6% (56.1, 81.7) in S1, 76.1% in S2 (65.6, 84.2), and 83.1% (73.5, 90.4) in S3. Compared with CoronaVac-S3, S1 and S2 showed significant differences (P<0.001 and P = 0.022, respectively). Within the S2 groups, infection inhibition percentage remained significantly higher in Covilo recipients than in CoronaVac recipients (P = 0.027).

For the Delta variant at T1, inhibition percentage in the Covilo subgroups were 24.6% (10.1, 42.8) for S1, 34.2% (25.0, 55.9) for S2, and 38.1% (21.5, 63.8) for S3, with S1 showing significant differences with S2 (P = 0.013) and S3 (P = 0.006). For CoronaVac, rates were 23.6% (12.6, 40.4) in S1, 29.8% (15.0, 38.7) in S2, and 24.2% (6.9, 44.3) in S3, with no differences among schedules (P = 0.535). However, among S2 and S3 recipients, Delta inhibition was significantly higher for Covilo than for CoronaVac (P = 0.008 and P = 0.001, respectively).

For Omicron, infection inhibition percentage remained 0.0% at T1 in all groups. At T1, WT infection inhibition percentage were higher than baseline (T0) in all interval groups (P<0.001) (**Figure 3**), with inhibition ranked higher for WT than for Delta, and Delta higher than Omicron (P<0.001) (**Figure 3**).

### Symptom analysis of COVID-19 and long COVID in Covilo recipients

Subsequent to the COVID-19 epidemic wave that occurred in China between December 2022 and April 2023, study recipients underwent follow-up and completed a structured questionnaire survey, 60/74 (81.1%) in S1, 57/72 (79.2%) in S2, and 55/74 (74.3%) in S3 reported COVID-19 symptoms. No significant differences were observed among the three groups (P = 0.606; **Table 4**). Muscular soreness was reported by 36 participants in S1 (48.6%), 28 in S2 (38.9%), and 21 (28.4%) in S3, with a significant difference among groups (P = 0.042). Runny nose occurred among two participants (2.7%) in S1, 11 (15.3%) in S2, and 11 (14.9%) in S3, with a significant difference among groups (P = 0.013). A symptom recovery time of ≥1 week was reported by 14 participants (23.7%) in S1, 20 (35.1%) in S2, and 16 (29.6%) in S3, with no significant between-group difference (P = 0.369).

**Table 4:** Symptoms of COVID-19 infection and long COVID among Covilo recipients.

Variable	S1(N = 74)	S2(N = 72)	S3(N = 74)	P
Symptoms of COVID-19 infection				
No	14(18.9)	15(20.8)	19(25.7)	0.606
Yes	60(81.1)	57(79.2)	55(74.3)	
Fever	29(39.2)	39(54.2)	30(40.5)	0.150
Fatigue	39(52.7)	32(44.4)	27(36.5)	0.155
Muscular soreness	36(48.6)	28(38.9)	21(28.4)	0.042
Cough	37(50.0)	28(38.9)	30(40.5)	0.351
Runny nose	2(2.7)	11(15.3)	11(14.9)	0.013
Diarrhea	5(6.8)	2(2.8)	0(0.0)	0.054
Nausea	3(4.1)	2(2.8)	1(1.4)	0.702
Vomiting	2(2.7)	2(2.8)	4(5.4)	0.736
Headache	15(20.3)	7(9.7)	7(9.5)	0.111
Pneumonia	1(1.4)	2(2.8)	1(1.4)	0.697
Symptom recovery time				
<1 week	46(76.7)	37(64.9)	38(70.4)	0.369
≥1 week	14(23.7)	20(35.1)	16(29.6)	
Symptoms of long COVID				
No	63(85.1)	49(68.1)	53(71.6)	0.039
Yes	11(14.9)	23(31.9)	21(28.4)	
Fatigue	7(9.5)	14(19.4)	17(23.0)	0.079
Sleepy	0(0.0)	2(2.8)	4(5.4)	0.128^a^
Headache/dizziness/migraine	1(1.4)	2(2.8)	5(6.8)	0.231^a^
Cough	1(1.4)	6(8.3)	2(2.7)	0.104
Discomfort in the pharynx	0(0.0)	5(6.9)	2(2.7)	0.035^a^

Data are number of participants (%), statistical analysis was conducted using the Chi-Squared Test and Fisher’s Exact Test. ^a^The following long COVID symptoms were each reported in three or fewer participants, with no significant differences between groups: altered/decreased taste/smell, gastrointestinal discomfort, decreased vision, shortness of breath, decreased exercise capacity, palpitations, chest pain, abnormal blood glucose, anemia, anxiety, sleep disorders, alopecia, and tinnitus/ear pain.

Symptoms of long COVID were reported by 11 participants (14.9%) in S1, 23 (31.9%) in S2, and 21 (28.4%) in S3, with a significant difference among the groups (P = 0.022; **Table 4**). Fatigue occurred in seven participants (9.5%) in S1, 14 (19.4%) in S2, and 17 (23.0%) in S3, with no significant difference among the groups (P = 0.079). Pharyngeal discomfort was only reported in the S2 and S3 subgroups, with five (6.9%) and two participants (2.7%), respectively, and a significant between-group difference (P = 0.035).

### Factors influencing symptoms

In multifactorial analysis, the main independent variables were incidence of COVID-19 infection and long COVID, while the dependent variables were sex, age, BMI and different vaccination schedules (**Table 5**). Chi-square test revealed a significant difference in incidence of COVID-19 infection by sex (P = 0.032), whereas no statistically significant intergroup variations were observed for age (P = 0.566), BMI (P = 0.397), or vaccination schedule (P = 0.505); a significant association between sex and the prevalence of long COVID (P = 0.001), while no such significance was found for age (P = 0.437), BMI (P = 0.959), or vaccination schedule (P = 0.051).

**Table 5:** Multifactorial analysis of COVID-19 infection symptoms and long COVID symptoms.

Variable	COVID-19 infection				Long COVID			
	Infection percentage	Chi-Squared test	Logistic test		Incidence	Chi-Squared test	Logistic test	
	n(%)	P	OR	95%CI	n(%)	P	OR	95%CI
Sex		0.032				0.001		
Female	88(84.6)		ref		38(36.5)		ref	
Male	84(72.4)		0.5	(0.2,0.9)	17(14.7)		0.3	(0.1,0.6)
Age, years		0.566				0.437		
60-69	111(79.9)		ref		34(24.5)		ref	
70-80	60(75.0)		0.8	(0.4,1.6)	20(25.0)		1.3	(0.7,2.6)
Body mass index (kg/m2)		0.397				0.959		
<24.0	113(79.6)		ref		35(24.6)		ref	
≥24.0	59(75.6)		0.7	(0.4,1.5)	20(25.6)		1.0	(0.5,1.9)
Different Vaccination Schedules		0.505				0.051		
S1	60(81.1)		ref		11(14.9)		ref	
S2	57(79.2)		0.8	(0.4,1.9)	23(31.9)		2.8	(1.2,6.5)
S3	55(74.3)		0.6	(0.3,1.4)	21(28.4)		2.2	(0.9,5.2)

Data presented as odds ratios (ORs) with 95% confidence intervals in parentheses, analyzed using binary logistic regression. Variables with P<0.1 in univariate analysis (sex, age, BMI, vaccine type, schedule) were included in binary logistic regression models for COVID-19 infection and long COVID. Model fit was assessed using the Hosmer–Lemeshow test (P>0.05 indicates good fit).

Binary logistic regression analysis identified, compared with females, males had significantly lower odds of experiencing COVID-19 infection (odds ratio [OR]=0.5; 95% CI: 0.2–0.9; P = 0.032) and long COVID-19 (odds ratio [OR]=0.3; 95% CI: 0.1–0.6; P = 0.001). No significant differences were observed between the 60–69- and 70–80-year age groups for COVID-19 infection (OR = 0.8; 95% CI: 0.4–1.6; P = 0.566), long COVID (OR = 1.3; 95% CI: 0.7–2.6; P = 0.437). Similarly, BMI (<24.0 vs. ≥24.0) showed no significant association with COVID-19 infection (OR = 0.7; 95% CI: 0.4–1.5; P = 0.397), long COVID (OR = 1.0; 95% CI: 0.5–1.9; P = 0.959). Different vaccination schedules showed no significant association with COVID-19 infection (S2: OR = 0.8; 95% CI: 0.4-1.9; P = 0.505) (S3: OR = 0.6; 95% CI: 0.3-1.4; P = 0.505). And for long COVID (S2: OR = 2.8; 95% CI: 1.2-6.5; P = 0.051) (S3: OR = 2.2; 95% CI: 0.9-5.2; P = 0. 0.051).

## Discussion

SARS-CoV-2 infection poses a disproportionately high threat to adults aged 60 years and above, with this population facing elevated risks of severe COVID-19, hospitalization and post-acute sequelae of COVID-19 (long COVID) due to age-related immune senescence, chronic comorbidities and impaired physiological function. Inactivated COVID-19 vaccines have been widely deployed for primary and booster immunization globally, and mounting evidence has confirmed their basic safety and immunogenicity in older adults (19). However, critical questions remain about the optimal booster interval, the durability of immune responses induced by different vaccination schedules, and the protective effects against COVID-19 symptoms and long COVID in this vulnerable group (27, 28). In this prospective cohort study of 450 elderly individuals in Zhejiang Province, we systematically evaluated the safety, humoral immune responses and clinical outcomes of two inactivated vaccines (CoronaVac and Covilo) under three distinct dosing schedules, and our findings provide valuable real-world evidence for refining booster vaccination strategies tailored to older adults.

The overall safety profile of the two inactivated vaccines was favorable in our study, with the incidence of vaccine-related adverse reactions ranging from 0.0% to 8.0% across all subgroups, and no severe adverse events reported. This is consistent with previous clinical trials and real-world studies of inactivated COVID-19 vaccines in elderly populations, where adverse reactions are predominantly mild and self-limiting (18). This aligns with observations from a homologous CoronaVac booster study in Yunnan, China, where injection-site pain was the most frequently reported local adverse event, and systemic reactions were rare (29). Systemic adverse reactions in our study were only observed in small subsets (Covilo-S2 with allergy, CoronaVac-S3 with fever), accounting for just 1.3% of participants in each affected subgroup, further confirming that inactivated vaccine boosters are well-tolerated by older adults (29).

Humoral immune responses showed distinct patterns across vaccination schedules and vaccine types (30, 31), with the 4–6 month interval third booster dose (S3) consistently inducing the strongest immune responses at 1 month post-vaccination (T1). At T1, the GMTs of neutralizing antibodies in both Covilo-S3 and CoronaVac-S3 were markedly higher than those in S1 and S2, with GMTs reaching 57.2 and 59.4, respectively-findings that support the notion that an extended interval between the second and third doses enhances the magnitude of the antibody response. Consistent with our findings, a study from Taiwan demonstrated that a fourth dose elicited anti-spike IgG titers 1.33-fold higher than peak levels after the third dose, further supporting that additional vaccine doses enhance humoral immunity in this population (31). A slight difference was observed in the S1 schedule, where CoronaVac induced a higher neutralizing antibody GMT (21.8) than Covilo (15.4), suggesting minor variations in the primary immune response of the two inactivated vaccines in the elderly. While neutralizing antibody titers declined progressively over the 12-month follow-up, the S3 schedule maintained the highest GMTs in both vaccine groups, with Covilo-S3 at 9.9 and CoronaVac-S3 at 10.0 at T4. Additionally, CoronaVac-S2 outperformed Covilo-S2 at T4 (8.0 vs. 5.1), indicating that vaccine-specific immune persistence differs even under the same dosing schedule. The dynamics of SARS-CoV-2-specific IgG antibodies (anti-S1+NP and anti-RBD) mirrored the neutralizing antibody results: the S3 schedule yielded the highest geometric mean concentrations (GMCs) at T1, with CoronaVac-S3 showing marginally higher anti-S1+NP IgG levels than Covilo-S3. For anti-RBD IgG, CoronaVac’s S2 and S3 schedules had significantly higher GMCs than S1, highlighting that a third booster dose is critical for inducing robust antibody responses against the receptor-binding domain-a key target for SARS-CoV-2 neutralization.

In terms of antibody responses against SARS-CoV-2 Delta and Omicron Variants, our pseudovirus-based neutralization assays revealed that the S3 schedule conferred the strongest inhibition of the wild-type (WT) SARS-CoV-2 strain and Delta variant at T1, with Covilo-S2 and S3 exhibiting superior inhibition compared to their CoronaVac counterparts for both variants. This is in line with a Hong Kong study that reported third-dose booster vaccination significantly enhanced neutralization activity against the ancestral strain and Delta variant in older adults (32). A third mRNA booster after two ChAdOx1-S doses significantly enhances neutralization against SARS-CoV-2 variants, according to a Taiwanese study on healthcare workers (33). A third dose of mRNA vaccine significantly boosts antibody responses in older care home residents and, for the first time, effectively elicits neutralizing capacity against the Omicron variant. Although antibody levels decline rapidly, T-cell cross-recognition remains well-preserved, highlighting its critical role in protecting older adults against immune-evasive variants (34). A study from Canada shows that a third dose of an mRNA vaccine significantly enhances humoral immune responses in older adults, bringing their antibody levels, functional activity, and neutralizing capacity against the Omicron variant to levels comparable to those observed in younger individuals (35). A study from Taiwan demonstrated that a fourth dose of mRNA vaccine significantly increased neutralizing activity against the D614G variant in older adults, with fold-rises ranging from 2.76 to 4.07; however, neutralization titers against the XBB.1.16 subvariant were 17- to 29-fold lower than those against D614G, highlighting the immune evasive nature of emerging Omicron sublineages (31). Although multiple real-world studies have shown that antibody levels gradually decline over time, inactivated vaccines can still maintain durable protection against severe disease, highlighting the role of immune memory (36–38).

Notably, all vaccination groups showed weakest inhibition of the Omicron variant at T1, a finding consistent with global research on inactivated COVID-19 vaccines. Omicron’s extensive mutations in the spike protein, particularly in the RBD region, enable it to evade neutralizing antibodies induced by vaccines targeting the ancestral strain, which explains the lack of effective neutralization observed in our study (39). The hierarchical inhibition of WT > Delta > Omicron across all groups further underscores the impact of viral evolution on the protective efficacy of first-generation inactivated vaccines, and the need for updated vaccines targeting circulating variants for this population (40).

Our clinical outcome data showed that the S2 and S3 schedule was associated with the lowest incidence of long COVID among recipients, with S1 having the highest rates-14.9% for long COVID in S1 compared to 31.9% in S2 and 28.4% in S3. Muscular soreness and runny nose were the most significantly different symptoms across schedules, with higher rates in S1, while the duration of symptom recovery did not differ notably. Multivariate logistic regression analysis identified male sex as a protective factor against COVID-19 infection rates and long COVID, with an odds ratio of 0.5 and 0.3, which is consistent with several international cohort studies reporting gender differences in COVID-19 clinical outcomes, possibly due to biological and immunological differences between males and females. Our findings also align with a large-scale study across the UK, Spain and Estonia that confirmed COVID-19 vaccination reduces the risk of long COVID, and extend this evidence by showing that optimized booster intervals further lower this risk in older adults (41). However, it is important to note that while booster vaccination reduced symptom severity and long COVID risk, it did not eliminate breakthrough infections-likely due to the widespread circulation of the Omicron variant during the study’s follow-up period, which evades vaccine-induced immunity, as reported in a Hong Kong study that found limited protection of CoronaVac and BNT162b2 boosters against Omicron infection in older adults (42).

This study has several notable limitations that should be acknowledged when interpreting its findings. First, our assessments of SARS-CoV-2 neutralizing activity were primarily centered on the ancestral strain and Delta variant, with extremely limited testing of the Omicron variant and its sublineages; as Omicron had become the dominant circulating strain during the study’s follow-up period, this gap weakens the clinical relevance of our results for the current COVID-19 epidemic landscape and prevents clear conclusions about the booster’s protective efficacy against this prevalent variant. Second, the research only quantified humoral immunity-related indices, such as neutralizing antibody titers and SARS-CoV-2-specific IgG levels, and did not evaluate cell-mediated immunity-an essential component of long-term immune protection induced by COVID-19 vaccines. The lack of cellular immunity data means we cannot fully and comprehensively elucidate the overall immune protection mechanism of inactivated COVID-19 vaccines in the elderly population. Third, while this was a prospective cohort study, local adjustments to COVID-19 vaccination policies during the long-term follow-up led to a certain rate of participant loss to follow-up; in addition, the inherent observational nature of the study design introduced potential confounding factors, including unaccounted differences in participants’ underlying chronic diseases and individual baseline immune status, which may have interfered with the accuracy of our study outcomes. Fourth, all study participants were recruited from elderly populations in Zhejiang Province, China, resulting in obvious geographical restriction; meanwhile, the age range of the subjects was limited to 60–80 years, with no inclusion of the oldest-old population over 80 years old.

In conclusion, our study demonstrates that a third booster dose of either inactivated vaccine significantly enhances immune responses in adults aged 60 years and older, particularly when administered 2–6 months after the second dose. This booster induces substantial increases in neutralizing antibody levels, thereby improving immune protection against SARS-CoV-2 and its variants; it also effectively reduces the symptoms, severity, and duration of COVID-19 infection, highlighting the critical role of vaccination in preventing long COVID.

## Materials and methods

### Study design and participants

**Figure 4:**
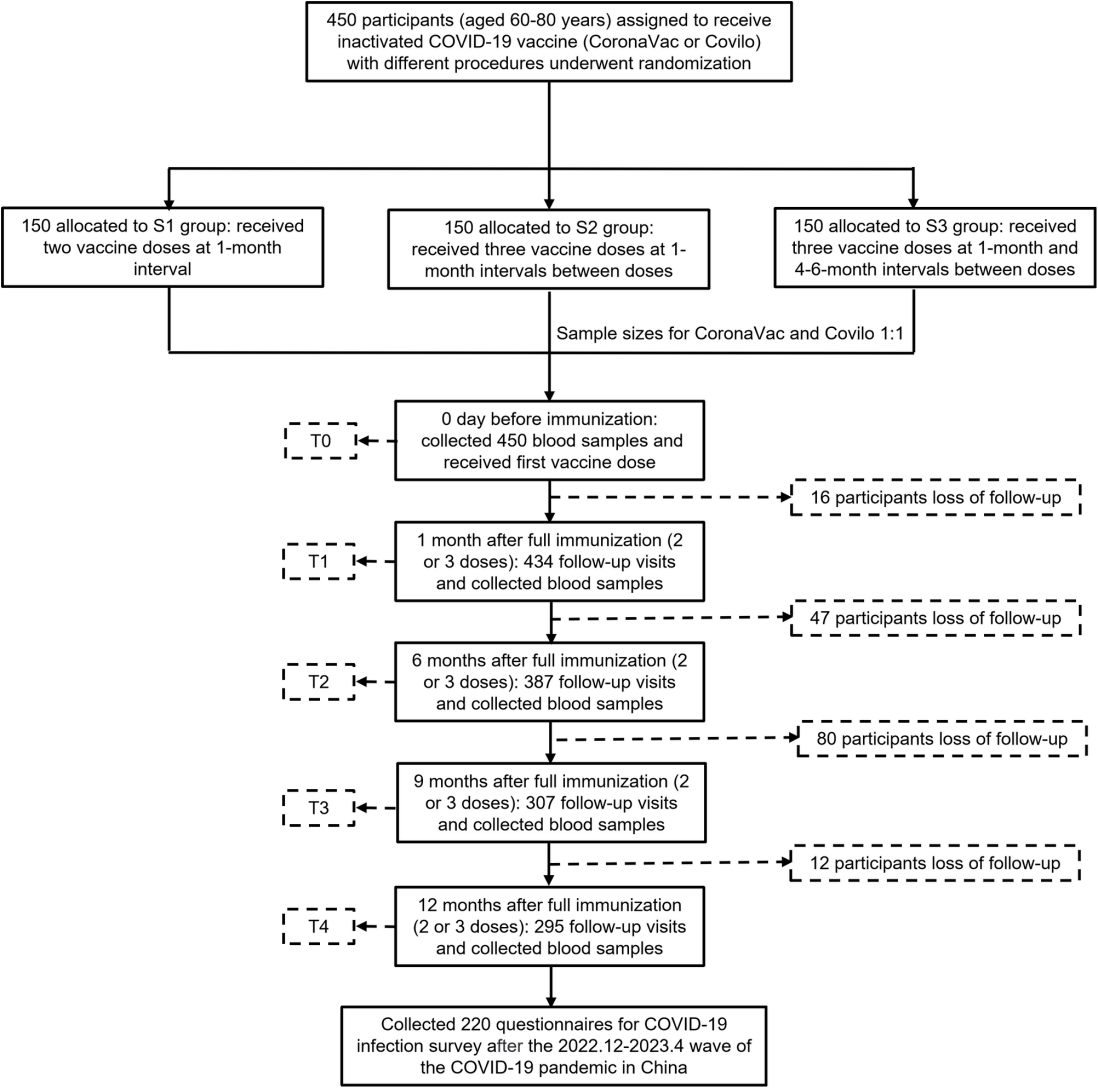
Study profile for the vaccination schedule and follow-up. A total of 450 participants aged ≥60 years were enrolled in this study and randomly assigned to three parallel groups (S1, S2, S3; 150 participants per group) to receive inactivated COVID-19 vaccines (CoronaVac and Covilo, administered in a 1:1 ratio) following distinct immunization schedules. The S1 group received two vaccine doses at a 1-month interval; the S2 group received three doses, with each dose administered at 1-month intervals; and the S3 group received three doses, with a 1-month interval between the first and second doses, and an interval of 4–6 months between the second and third doses. Venous blood samples were collected from all participants at five predefined time points: prior to the first dose (T0); and at 1 (T1), 6 (T2), 9 (T3), and 12 (T4) months after completion of the assigned vaccination schedule. As local COVID-19 vaccination policies were adjusted, some or all participants were mandated to receive a third vaccine dose at the T2 time point; consequently, a portion of participants were lost to follow-up.

This study enrolled a total of 450 participants aged ≥60 years, who were randomly assigned to three groups (S1, S2, S3, with 150 participants in each) to receive inactivated COVID-19 vaccines according to different schedules: the S1 group received two doses (1-month interval), the S2 group received three doses (1-month intervals between doses), and the S3 group received three doses (1-month interval between the first and second doses, and 4–6 months between the second and third doses) (**Figure 4**).

Two COVID-19 inactivated vaccines were used in this study, CoronaVac and Covilo (1:1 ratio). Administered by appropriately trained staff at the trial sites, participants received vaccine via intramuscular injection into the upper arm. Participants were observed for at least 30 min after vaccination. Then, venous blood was collected from recipients at five points: before the first dose (T0); 1 month (T1) after all doses; 6 months (T2) after all doses; 9 months (T3) after all doses; and 12 months (T4) after all doses. Safety endpoints were followed up by trained staff on-site or by phone for the first 7 days.

A sample size of 450 participants (150 in each groups) was calculated assuming a baseline seroconversion rate of 70% in the control group (S1), a 20% relative increase in the intervention groups (S2/S3), 80% statistical power, and a two-sided α of 0.05, with a 10% adjustment for potential loss to follow-up.

Throughout the study period, all participants were closely monitored, and none received any additional COVID-19 vaccines after completing their assigned schedules. In addition, all enrolled participants had no history of SARS-CoV-2 infection at baseline, and regular follow-ups confirmed that their first infection occurred after December 2022, thereby minimizing potential confounding from prior infection or additional vaccination.

### Vaccination safety assessment

This study adopted a combined approach of active and passive surveillance to assess vaccine safety profiles. All participants were required to undergo a 30-minute post-vaccination observation period, during which healthcare personnel documented any emerging adverse events and provided participants with diary cards accompanied by standardized completion instructions. The research team conducted active telephone follow-ups on Day 7 (with the vaccination day designated as Day 0) to gather health status updates and verify the accurate completion of diary cards. Participants were instructed to promptly report any suspected adverse events following immunization (AEFI), which were managed in strict accordance with the National Guidelines for AEFI Surveillance. Surveillance covered both local reactions (including induration, erythema, and swelling) and systemic reactions (including fever and allergic reactions), with symptom severity evaluated using the Grading Criteria for Adverse Events in Preventive Vaccine Clinical Trials. Administration of non-emergency vaccines was required to be separated from COVID-19 vaccination by an interval of at least two weeks; this requirement was waived for emergency vaccines (e.g., rabies vaccine). A comprehensive safety assessment was accomplished via systematic record-keeping using both diary cards and standardized follow-up forms.

### SARS-CoV-2-specific IgG assay

A commercial iFlash-2019-nCoV NAb assay kit (Shenzhen YHLO Biotech Co., Ltd., Shenzhen, China) was used to quantify the levels of IgG antibodies against the SARS-CoV-2 spike glycoprotein (S) and nucleocapsid protein (N) via chemiluminescence immunoassay (CLIA). According to the manufacturer’s instructions, titers of ≥ 10.0 AU/mL was defined as positive (or reactive). SARS-CoV-2 receptor-binding domain (RBD)-specific IgG were detected using a commercial ELISA kit (Bioscience Biotech Co., Ltd., Chongqing, China). The positive cutoff values for RBD-specific IgG were defined as titers of ≥ 1.0 AU/mL. All assays were performed strictly in accordance with the manufacturers’ instructions (7).

### Pseudovirus-based neutralization test

Serum samples, positive controls, and negative controls were first diluted 50-fold in PBS. A 96-well plate was labeled corresponding to sample identifiers. Then, 50 μL of diluted pseudovirus solution (WT, Delta, or Omicron variant) was added to each well, and the resulting serum-virus mixtures were incubated at 37 °C in a 5% CO_2_ incubator for 1 hour. BHK-21-ACE2 cells were harvested using standard cell collection protocols and resuspended to a concentration of 2×10^5^ cells/mL. Subsequently, 100 μL of the cell suspension was added to each well, followed by further incubation at 37 °C in a 5% CO_2_ incubator for 48 hours. Following 48 h of incubation, the number of GFP-positive (GFP^+^) cells in each well was quantified using a microplate reader (Tecan, SparkCyto). The neutralizing antibody inhibition percentage of each sample was calculated based on the fluorescence intensity readouts. The cell lines used in this study were obtained from the National Collection of Authenticated Cell Cultures (NCACC), Chinese Academy of Sciences.

### Microneutralization assays

Neutralizing antibody titers against live SARS-CoV-2 virus strain (19nCoVCDC-Tan-HB01) were quantified using the micro virus neutralization test (MVNT). Briefly, serum samples were heat-inactivated at 56 °C for 30 min, followed by two-fold serial dilution in 96-well plates. Diluted sera were mixed with SARS-CoV-2 working virus containing 50% tissue culture infective dose (TCID^50^) and co-incubated at 37 °C for 1 h. Subsequently, the serum-virus mixtures were transferred to 96-well plates pre-seeded with VeroE6 cells and further incubated at 37 °C for an additional hour. After a 3-day infection period, cytopathic effect (CPE) in VeroE6 cells was microscopically examined. Neutralizing antibody endpoint titers were calculated using either the Reed-Muench (43) or Spearman-Kärber (44) method when a 50% reduction in viral replication was observed. A neutralizing antibody titer of ≥ 1:8 was defined as seropositive. For data analysis, samples with titers below this cutoff value were assigned a nominal titer of 1:2.

### COVID-19 infection data collection

The COVID-19 infection questionnaire used in this study was designed based on previous research and finalized after panel discussions with multiple medical experts. The questionnaire consists of three sections: baseline characteristics, COVID-19 vaccination history, and SARS-CoV-2 infection status (including long COVID information). Collected baseline characteristics included study ID, follow-up outcomes, underlying diseases, and other medical conditions. For COVID-19 infection status, participants were asked: “Have you ever been diagnosed with COVID-19 or experienced COVID-like symptoms after November 2022?” Those who responded affirmatively were required to provide additional details including infection/symptom onset time and specific symptoms. Prior to completing the questionnaire, all eligible participants received information about the study’s purpose, risks, and benefits. The survey was then administered through the Wenjuanxing platform (www.wjx.cn).

### Statistical analysis

Participants were randomly assigned to three schedules using a block randomization method (block size=6) generated by SAS 9.4 software, and allocation was concealed via sequentially numbered, opaque sealed envelopes. Within each schedule group, participants were further allocated to CoronaVac or Covilo at a 1:1 ratio using simple randomization. Demographic Characteristics, Adverse events, and so on of the participants were collected for each vaccination recipient. The categorical variables such as sex, age, and so on, are presented as numbers (percentages), the data not following the normal distribution were expressed as the median (quartile) [M (P25, P75)]. We used Student’s t-test and one-way analysis of variance with Tukey’s multiple comparisons test to analyze log-transformed antibody titers and categorical data. The Wilcoxon rank-sum test and Kruskal-Wallis test were utilized for non-normally distributed data. The chi-square test and Fisher’s exact test were performed to analyze categorical data. A logistic regression model (enter method) was used for multivariate analysis. All statistical tests were two-sided, and P-values <0.05 were considered statistically significant. All analyses were conducted using SAS 9.4 (SAS Institute Inc., Cary NC, USA) and Prism software version 9.0.2 (GraphPad Software, Inc., San Diego CA, USA) software.

## Data Availability

The original contributions presented in the study are included in the article/supplementary material. Further inquiries can be directed to the corresponding authors.
